# Biological Advances and Current Challenges for Pediatric Rhabdomyosarcoma

**DOI:** 10.3390/cancers18060888

**Published:** 2026-03-10

**Authors:** Katie E. Hebron, Patience Odeniyide, Yun Wei, Berkley E. Gryder, Frederic G. Barr, Dana L. Casey, Eleanor Y. Chen, Brian D. Crompton, Filemon S. Dela Cruz, Adam D. Durbin, Heide L. Ford, Susanne A. Gatz, Mark E. Hatley, Anton G. Henssen, Simone Hettmer, Peter J. Houghton, Genevieve C. Kendall, Javed Khan, Philip J. Lupo, Anand G. Patel, Silvia Pomella, Rossella Rota, Marco Schito, Reineke A. Schoot, Jack F. Shern, Benjamin Z. Stanton, Elizabeth A. Stewart, Cathy A. Swindlehurst, Craig J. Thomas, Christopher R. Vakoc, Angelina V. Vaseva, Rajkumar Venkatramani, Leonard H. Wexler, Jason T. Yustein, Sharon Hammond, Christine M. Heske, David M. Langenau, Corinne M. Linardic, Myron S. Ignatius, Marielle E. Yohe

**Affiliations:** 1 Laboratory of Cell and Developmental Signaling, National Cancer Institute at Frederick, National Institutes of Health, Frederick, MD 21701, USA; kehebron@purdue.edu; 2 Basic Medical Sciences, College of Veterinary Medicine, Purdue University, West Lafayette, IN 47906, USA; 3 Division of Pediatric Oncology, The Sidney Kimmel Comprehensive Cancer Center, Johns Hopkins University School of Medicine, Baltimore, MD 21287, USA; pobasaj1@jhmi.edu; 4 Molecular Pathology Unit, Massachusetts General Research Institute, Harvard Medical School, Charlestown, MA 02129, USA; yun.wei@utsouthwestern.edu (Y.W.);; 5 Krantz Family Center for Cancer Research, Massachusetts General Hospital, Charlestown, MA 02129, USA; 6 Division of Hematology and Oncology, Department of Pediatrics, UT Southwestern Medical Center, Dallas, TX 75390, USA; 7 Department of Genetics and Genome Sciences, Case Western Reserve University School of Medicine, Cleveland, OH 44106, USA; 8 Laboratory of Pathology, National Cancer Institute, Bethesda, MD 20892, USA; 9 Department of Radiation Oncology, University of North Carolina School of Medicine, Chapel Hill, NC 27514, USA; 10 Department of Laboratory Medicine and Pathology, University of Washington, Seattle, WA 98115, USA; 11 Department of Pediatric Oncology, Dana-Farber Cancer Institute, Boston, MA 02215, USA; 12 Pediatrics, Harvard Medical School, Boston, MA 02115, USA; 13 Boston Children’s Hospital, Boston, MA 02115, USA; 14 Cancer Program, Broad Institute of Harvard and MIT, Cambridge, MA 02142, USA; 15 Department of Pediatrics, Memorial Sloan Kettering Cancer Center, New York, NY 10065, USA; delacrf1@mskcc.org (F.S.D.C.);; 16 Division of Molecular Oncology, Department of Oncology, St. Jude Children’s Research Hospital, Memphis, TN 38105, USAmark.hatley@stjude.org (M.E.H.); 17 Department of Pharmacology, University of Colorado Anschutz Medical Campus, Aurora, CO 80045, USA; 18 Department of Cancer and Genomic Sciences, School of Medical Sciences, College of Medicine and Health, University of Birmingham, Birmingham B15 2TT, UK; 19 Department of Paediatric Oncology, Women’s and Children’s NHS Foundation Trust, Steelhouse Ln, Birmingham B4 6NH, UK; 20 Charité-Universitätsmedizin Berlin, Corporate Member of Freie Universität Berlin and Humboldt-Universität zu Berlin, Germany, Department of Pediatric Hematology and Oncology, Augustenburger Platz, 13353 Berlin, Germany; 21 University Medicine Halle, Pediatrics, Martin Luther University Halle, 06108 Halle, Germany; 22 Greehey Children’s Cancer Research Institute, UT Health San Antonio, San Antonio, TX 78229, USA; 23 Department of Molecular Medicine, UT Health San Antonio, San Antonio, TX 78229, USA; 24 Center for Childhood Cancer Research, Nationwide Children’s Hospital, Columbus, OH 43205, USA; 25 Department of Pediatrics, The Ohio State University College of Medicine, Columbus, OH 43210, USA; 26 Genetics Branch, National Cancer Institute, National Institutes of Health, Bethesda, MD 20892, USA; 27 Division of Hematology/Oncology, Department of Pediatrics, Emory University, Atlanta, GA 30322, USA; 28 Department of Oncology, St. Jude Children’s Research Hospital, Memphis, TN 38105, USA; 29 Department of Developmental Neurobiology, St. Jude Children’s Research Hospital, Memphis, TN 38105, USA; 30 Department of Hematology and Oncology, Cell and Gene Therapy, Bambino Gesù Children’s Hospital, IRCCS, 00165 Rome, Italy; silvia.pomella@opbg.net (S.P.);; 31 Department of Clinical Sciences and Translational Medicine, University of Rome Tor Vergata, 00133 Rome, Italy; 32 UGenome AI, Tucson, AZ 85614, USA; 33 Princess Máxima Center, 3584 CS Utrecht, The Netherlands; 34 Pediatric Oncology Branch, National Cancer Institute, National Institutes of Health, Bethesda, MD 20892, USA; 35 Department of Biological Chemistry & Pharmacology, The Ohio State University College of Medicine, Columbus, OH 43210, USA; 36 Katalytic Therapeutics, San Diego, CA 92126, USA; 37 Division of Pre-Clinical Innovation, National Center for Advancing Translational Sciences (NCATS), National Institutes of Health, Rockville, MD 20850, USA; 38 Cold Spring Harbor Laboratory, New York, NY 11724, USA; 39 Department of Pediatrics, School of Medicine, Oregon Health and Science University, Portland, OR 97239, USA; vaseva@ohsu.edu; 40 Department of Cell, Developmental, and Molecular Biology, School of Medicine, Oregon Health and Science University, Portland, OR 97239, USA; 41 Department of Pediatrics, Texas Children’s Hospital, Baylor College of Medicine, Houston, TX 77030, USA; 42 Winship Cancer Institute and Aflac Cancer and Blood Disorders Center, Emory University, Atlanta, GA 30322, USA; 43 Summer’s Way Foundation, Centennial, CO 80016, USA; 44 Harvard Stem Cell Institute, Harvard Medical School, Cambridge, MA 02115, USA; 45 Division of Hematology-Oncology, Department of Pediatrics, Duke University School of Medicine, Durham, NC 27701, USA; corinne.linardic@duke.edu

**Keywords:** pediatric sarcoma, cells of origin, animal models, tumor heterogeneity, skeletal muscle malignancy

## Abstract

Despite comprehensive and multi-modal therapy, outcomes for children and adolescents with rhabdomyosarcoma (RMS) have plateaued over the past four decades. This is not for a lack of progress in the basic and translational studies of RMS. Indeed, advances in animal models and/or patient tissue sample acquisition and analysis have improved our understanding of RMS biology. Large-scale sequencing efforts have generated transcriptomic, genomic, and epigenomic datasets that highlight the heterogeneity of RMS and have the potential to improve prognostication and the application of precision medicine in patients with RMS. However, few of these discoveries have been clinically translated, and limitations to the accessibility, uniformity, and application of these new models and datasets hinder their utility. Here, we discuss how advances in understanding RMS biology, optimization of preclinical models, and strategies for translating basic science discoveries to the clinic can potentially improve outcomes for patients with RMS.

## Simple Summary

Outcomes for children and adolescents with rhabdomyosarcoma (RMS), the most common soft tissue sarcoma of childhood, have not improved over the last four decades, despite discoveries made in basic and translational studies of RMS. In this review, we synthesize recent insights into RMS biology including emerging evidence for the possibility of diverse cell(s) of origin, advances in describing and modelling intra- and intertumoral heterogeneity, and emerging mechanisms of cell-state plasticity. We discuss how the field can leverage these discoveries in combination with large-scale datasets and collaborative initiatives to potentially improve clinical outcomes for patients with RMS.

## 1. Introduction

Rhabdomyosarcoma (RMS) is the most common soft tissue sarcoma of childhood, with approximately 350 newly diagnosed cases in the United States annually [1,2,3,4]. Patients with relapsed, refractory, or metastatic disease have a dismal prognosis, with 6-month event-free survival rates under 20% [5]. Current therapies are associated with short-term and frequently lifelong morbidities. Thus, more effective and less toxic therapies are needed to improve outcomes for high-risk patients.

There are four histologic subtypes of RMS: alveolar (ARMS), embryonal (ERMS), spindle cell/sclerosing (SCRMS), and pleomorphic (PRMS) (Table 1) [6]. PRMS generally affects patients aged 40–70 years and will not be discussed in this review [3]. ERMS is the most common RMS subtype and occurs in patients in a bimodal age distribution, predominantly those aged 0–4 and 14–18 years [3,7,8]. ARMS, the second most common subtype, typically occurs in late childhood and adolescence and is generally associated with poorer outcomes than ERMS. Most ARMS tumors have chromosomal translocations involving chromosome 13 and chromosomes 2 or 1, resulting in the expression of the fusion oncogenes *PAX3::FOXO1* or *PAX7::FOXO1* [4,9,10]. In addition, other rare fusion variants, including *PAX3::NCOA1/INO80D* and *PAX3::MAML3*, have been reported in ARMS tumors [11,12,13]. In current risk stratification, these tumors are molecularly classified as fusion-positive RMS (FP-RMS), which is the strongest predictor of poor prognosis after metastatic disease [14,15]. Additional fusions such as *EWSR1::NF2* and *FGFR1::ANK1* have been discovered in RMS tumors with mixed or undefined histology, but their prognostic value has yet to be defined [16,17,18]. Some ARMS tumors do not have the *PAX3::FOXO1* or *PAX7::FOXO1* fusion oncogenes and are considered fusion-negative RMS (FN-RMS).

Several genetic lesions have been discovered in tumors with SCRMS histology. SCRMS diagnoses in infancy are typically associated with *NCOA2::VGLL2* fusions and have a favorable prognosis, whereas SCRMS in young adult patients commonly harbor a *MYOD1^L122R^* mutation and have an extremely poor prognosis [19,20,21,22,23]. Ultra-rare intraosseous SCRMS tumors associated with *EWSR1::TFCP2*, *FUS::TFCP2*, or *MEIS1::NCOA2* fusions also occur in the adolescent and young adult population and present a significant diagnostic and treatment challenge with >60% mortality [24].

Decades of laboratory and clinical research have enhanced our understanding of RMS biology, significantly improved risk stratification, and refined the application of established chemotherapy and radiotherapy protocols. However, these efforts have not yet led to the identification of novel therapies for patients with RMS. Several challenges continue to limit our understanding of RMS biology and the translation of basic science discoveries into therapies, including cell(s)-of-origin identification, intra- and intertumoral heterogeneity, cell plasticity, and disease rarity. While it is widely accepted that RMS tumors have features of poorly differentiated skeletal muscle cells, several animal models support a non-muscle cell of origin [25,26,27,28,29,30]. High levels of heterogeneity (e.g., different driver oncogenes) and plasticity (e.g., trans-differentiation) in RMS likely contribute to the lack of consensus. However, the contribution of potentially divergent cells of origin to tumor etiology, prognostication, and therapeutic response has not been elucidated. Heterogeneity and plasticity also factor into challenges with preclinical modeling, which can hamper clinical translation. Large-scale next-generation sequencing efforts show significant intra- and intertumoral heterogeneity that is difficult to replicate preclinically. Challenges in preclinical study design are exacerbated by plasticity in tumor cell subpopulations that allow cells to transition from actively dividing cells that are sensitive to anticancer agents towards more indolent states that pose therapeutic challenges [31,32,33,34,35]. Finally, the rarity of RMS presents several challenges to the translation of basic discoveries, including dividing the already small number of patients by molecular subtypes, which results in even smaller groups of patients to study, limitations on the collection and number of patient tumor samples, slow accrual to clinical trials, and limited interest from the pharmaceutical industry to develop drugs for small populations. Here, we discuss how advances in RMS biology, modeling, and shared resources by the research community, such as omics datasets, can be leveraged to address these challenges. This review focuses on the following lingering questions:

1. What are the cells of origin of RMS?
2. How can preclinical models be improved to more accurately represent RMS heterogeneity and plasticity?
3. How can sequencing technologies be leveraged to gain a better understanding of RMS biology and predict clinical response to therapies?
4. What are the challenges impeding the clinical translation of basic discoveries in RMS, and how can they be addressed?

## 2. What Are the Cells of Origin of RMS?

### 2.1. Muscle Lineage

Early histopathological studies suggest that both ERMS and ARMS are muscle lineage-derived, with most RMS tumors expressing Desmin, a marker of mature skeletal muscle. Myogenic lineage transcription factors Myogenic Differentiation 1 (MYOD1), Myogenic Factor 5 (MYF5), and Myogenin (MYOG) are enriched in these tumors by immunohistochemistry. These markers are highly expressed during early muscle development but repressed in mature fused muscle. Additionally, rhabdomyoblasts histologically resemble primitive mesenchymal cells with varying degrees of myogenic differentiation, from round and spindle-like cells to fused muscle fibers with occasional striations.

The data supporting a myogenic cell of origin extends past descriptive similarities [8,36]. CRISPR-dropout screens have identified myogenic regulator factors (MRFs) such as *MYOD1*, *MYOG*, *MYF5*, and *PAX7* as top dependency genes in FP-RMS and FN-RMS cell lines (DepMap, https://depmap.org/portal/) (accessed on 26 February 2026) [37,38]. MYF5 and MYOD1 each transcriptionally regulate a specific subset of muscle genes in RMS cells and are each indispensable for sustained tumor growth. These transcriptional factors are critical regulators of muscle development that are not expressed in other tissue types [37]. In addition, insights gained from the creation of genetically engineered animal models lend support to the hypothesis that RMS may be derived from a muscle precursor cell, as muscle lineage-specific expression of the driver oncogenes has resulted in the generation of RMS-like tumors. For example, orthotopic tumors histologically and transcriptionally resembling FN-RMS have been derived from human or mouse myoblasts transformed by oncogenic *RAS* expression combined with *TP53/RB* loss of function and telomere stabilization [39,40]. Homozygous conditional activation of *Pax3::Foxo1* fusion in *Myf6*-expressing murine skeletal muscle cells, as well as the stable expression of PAX3::FOXO1 along with MYCN amplification, p16^INK4A^ down-regulation, and telomere stabilization in human myoblasts, initiated the formation of spontaneous and orthotopic tumors, respectively, in mice with histological and immunohistochemical resemblance to human ARMS [41,42,43]. Furthermore, tumors from zebrafish ERMS models have activated satellite cell-like transcriptomic profiles [44].

Recent single-cell RNA sequencing (scRNAseq) studies have provided another level of insight into the cells of origin of RMS by comparing expression profiles of RMS subpopulations with subpopulations from human or mouse embryonal/fetal muscle. These data identified progenitor, proliferative, differentiated, ground, and apoptotic cell subpopulations in FN-RMS and FP-RMS patient- and cell line-derived xenograft samples [31,32]. Interestingly, these studies failed to identify a significant similarity in gene expression signatures with satellite cells, which were thought to be the cell of origin. FN-RMS progenitor cells map to human skeletal muscle mesenchymal cells, for which no mouse or other animal counterpart has been defined [31,35]. FP-RMS maps to an even narrower window during muscle development, the transition stage between embryonic and fetal muscle development [31]. Yet, some researchers argue that muscle-lineage commitment is not necessary for the transformation of malignancy to RMS. Tissue that precedes the embryonic or fetal muscle could be a cell of origin for rhabdomyosarcomas. For example, expression of *PAX3::FOXO1* or *PAX7::FOXO1* in mesenchymal stem cells formed ARMS-like tumors in mice, supporting the hypothesis of a cell of origin that may precede myogenic lineage commitment [45]. This idea is also supported by scRNAseq studies that mapped the putative cancer stem cell subpopulation to mouse mesodermal cells [33]. While initially thought to be expressed postnatally [42], *Myf6* is also expressed during embryonic myogenesis (reviewed in [46]). Therefore, the *Myf6*-driven FP-RMS genetically engineered mouse model (GEMM) also indicates that events during very early embryogenesis may underlie FP-RMS [41]. Cumulatively, the essentiality of MRFs, muscle lineage-derived animal models, satellite cell-like morphology and transcriptional profiles, and expression profiles elucidated with scRNAseq provide strong evidence for a muscle lineage cell of origin.

### 2.2. Non-Muscle Lineage

Cell plasticity contributes to ambiguity in the RMS cell(s)-of-origin. Other cell types, including neuronal and endothelial cells, have been shown to adopt skeletal muscle properties when RMS driver mutations are expressed [25,26,27,29,30,45]. Supporting a potential neuronal cell of origin in FP-RMS, *in ovo* expression of *PAX3::FOXO1* or *PAX7::FOXO1* in chicken embryo neural cells induced their trans-differentiation to FP-RMS-like cells with myogenic characteristics [30]. Furthermore, in a *PAX3::FOXO1* transgenic zebrafish model, *HES3* (an essential regulator of neural crest differentiation) cooperated with *PAX3::FOXO1* to promote aggressive FP-RMS-like tumors [29].

Evidence in support of an endothelial cell of origin comes from mouse models with Hedgehog pathway activation or *Pax3::Foxo1* expression in aP2-expressing cells, which formed RMS tumors with histological and transcriptomic resemblance to FN-RMS and FP-RMS, respectively [25,27]. While aP2 was initially thought to mark cells of the adipose lineage, lineage tracing demonstrated that the tumors arose from *Kdr*-expressing endothelial precursors that are exclusive to the murine head and neck region, raising the question of whether the primary tumor site, a prognostic indicator in RMS [47], is a result of the cell of origin [25,26]. Furthermore, PAX3::FOXO1 expression in *Tek* (*Tie2*)-expressing endothelial cells gave rise to FP-RMS in the head and neck of GEMMs. PAX3::FOXO1 expression in both human umbilical vein endothelial cells (HUVECs) and human induced pluripotent stem cells (iPSCs) under endothelial-directed differentiation gave rise to FP-RMS when xenografted into immunocompromised mice, further highlighting the potential for cells of origin outside of the muscle lineage [27]. However, expression of *Pax3::Foxo1* in *Myf6*-expressing cells of myogenic lineage also induced predominantly head and neck RMS when combined with Hippo pathway inactivation and *Ink4a/ARF* loss in an FP-RMS GEMM [48]. Notably, KRAS^G12D^ expression in aP2-expressing cells resulted in tumors resembling angiosarcoma rather than RMS, suggesting that the potential of an endothelial cell of origin may be restricted to specific genotypes [25,26].

Understanding the potential cell(s) of origin in RMS is clinically relevant, as it may have therapeutic implications. For example, *PAX3::FOXO1* fusion oncogene expression within different cells of origin (satellite cells vs. myoblasts) produces ARMS-like tumors that respond differently to pharmacological perturbations [49]. Specifically, myoblast-derived ARMS cells are more susceptible to CDK9 and CDK4/6 inhibitors than satellite cell-derived ARMS cells in vitro. It has also been demonstrated that the progenitor cell subpopulations in FN-RMS map to skeletal muscle mesenchymal stem cells, which are quiescent cells that do not respond to chemotherapeutic agents [31,33,35]. In summary, the cell of origin of RMS remains an elusive question. While single-cell transcriptomics analysis validates its similarity to human embryonic or fetal muscle lineage, studies in GEMMs, zebrafish, and chicken embryos suggest the possibility of non-muscle lineage. Given the complexity and heterogeneity of the disease, it is possible that RMS can arise from multiple cells of origin. These putative cells of origin for rhabdomyosarcoma contribute to another layer of intertumoral heterogeneity and may provide clues for future precision medicine.

## 3. How Can Preclinical Models Be Improved to More Accurately Represent RMS Heterogeneity and Plasticity?

Accurately modeling RMS is essential for successfully translating basic and preclinical discoveries [50,51]. However, this remains challenging. Fusion status, mutational profile, site of disease, age at occurrence, and cell of origin are all potential sources of heterogeneity in RMS. These factors also influence prognosis and response to therapy [52]. Therefore, it is imperative that the models used in preclinical studies to evaluate the therapeutic potential of basic discoveries accurately reflect the heterogeneity of RMS and that model selection is guided by the clinical context in which a therapeutic intervention will be applied. Recent advances in genetically engineered FP- and FN-RMS, xenograft, and organoid models (Table 2) have brought the field closer to accurately modeling RMS, which may improve the ability to translate basic science discoveries. This section will cover advances in the representation of genetic lesions and patient demographics observed in RMS samples.

### 3.1. RAS Pathway Models

RAS pathway mutations are observed in the majority of FN-RMS [53,54]. They are considered driver mutations critical for FN-RMS development [55] and block myoblast differentiation via increased MEK/ERK signaling [44,56]. Despite the frequency of these mutations, RAS pathway-targeted agents have yet to show clinical benefit in patients with RMS [57]. Thus, additional studies, including more accurate modeling of RAS pathway mutations, are necessary to develop and test RAS-directed therapies, which may provide a less morbid and more effective alternative to cytotoxic chemotherapy. While activating mutations in all three RAS isoforms are observed in RMS, NRAS mutations, especially at amino acid Q61, are the most common [13,58]. However, most current genetically modified RMS models utilize KRAS^G12X^ mutations, which are less frequent [13,39,44,58,59,60,61,62,63,64]. RMS models driven by alterations in other RAS pathway genes, including *HRAS*, *NRAS*, and *NF1*, are available but underutilized [40,65,66,67,68]. Not all RAS alterations respond similarly to RAS-pathway targeting agents, especially with the advent of RAS isoform- and mutation-specific drugs [56,69]. Therefore, investigators should carefully consider including preclinical models that represent a variety of RAS isoforms and mutations when evaluating the efficacy of RAS pathway-targeting agents in RMS.

Currently available RMS cell lines and animal models accurately reflect the observation that RAS mutations, although considered drivers, frequently co-occur with additional mutations [58]. However, the diversity of secondary mutations is limited in both cell lines and genetically engineered models. *TP53* alterations occur in almost all human FN-RMS cell lines, including *RAS*-mutant FN-RMS cell lines, and *TP53* loss or mutation and *Ink4A/ARF* loss are the most common co-occurring mutations used to overcome *RAS* oncogene-induced senescence in animal models [39,44,62,63,64]. However, *TP53* and *Ink4A/ARF* account for a minority of the many mutations that co-occur with RAS pathway alterations in patients, undermining the potential fidelity of these models to represent the human disease [58]. *BCOR* and *NF1* alterations, the most common co-occurring mutations in human RAS-mutant FN-RMS [58], have yet to be modeled in genetically engineered animals, although some PDX (patient-derived xenograft) models express them (Table 3). Conducting preclinical evaluations using models that include secondary alterations reflective of the biology observed in patient samples is critical as they are more useful for identifying clinically relevant biomarkers of response [70,71,72]. Because several adult clinical and pediatric preclinical studies showed limited and short-lived responses to single-agent RAS pathway inhibitors (reviewed in [73]), accurately modeling tumor heterogeneity in RAS pathway-driven RMS is even more critical to identifying novel combination therapies.

**Table 2:** Genetically engineered animal models of rhabdomyosarcoma grouped by the subtype of human RMS that they most closely resemble.

Subtype	Organism	Genotype	Cell of Origin	Model Notes	Ref
FN-RMS	Mouse	HGF/SF; Ink4a/Arf^−/−^	Satellite cells suspected	Multifocal trunk and limb tumors with lung metastases, tumors observed in non-skeletal muscle organs	[74]
		M-Cre-Trp53^−/−^	Hypaxial myoblasts	Tumors in extremities, trunk, face with >60% metastasis to local lymph nodes and lungs	[75]
		M-Cre-Trp53^−/−^; Ptch1^+/−^	Hypaxial myoblasts	Tumors in extremities, trunk, face with >60% metastasis to local lymph nodes and lungs	[75]
		Myf5-Cre-Trp53^−/−^; Ptch1^+/−^	Myoblasts, satellite cells, brown adipose precursors	Tumors in extremities, trunk, face with >60% metastasis to local lymph nodes and lungs	[75]
		Myf6-Cre-Trp53^−/−^; Ptch1^+/−^	Differentiating myoblasts	Tumors in extremities and trunk with >60% metastasis to local lymph nodes and lungs	[75]
		Pax7^CE/+^; LSL-Kras^G12D/+^; Trp53^Fl/Fl^	Satellite cells	Tumors in body wall, extremities, head and neck, and subcutaneous, 62% UPS	[62]
		aP2-Cre; SmoM2	Endothelial	Exclusive to head and neck	[25]
		aP2-Cre; SmoM2; Cdkn2a^Fl/Fl^	Endothelial	Exclusive to head and neck	[25]
		MyoD1-Cre; KRas^G12D^; Trp53^R172H/+^	Embryonic progenitors	Tumors develop in limbs, trunk, pelvis, head, and neck; metastasis (mostly lung) in 10–20% of animals	[63]
	Zebrafish	rag2-KRAS^G12D^	Activated satellite cells	Dorsal lateral muscle with invasion to intestine, liver, kidneys, testes	[44]
		cdh15-KRAS^G12D^	Satellite cells, differentiating myoblasts	Spindle cell morphology	[61]
		mylz2-KRAS^G12D^	Differentiated muscle	Differentiated hypocellular tumors with vacuolated cells	[61]
FP-RMS	Mouse	Myf6-Cre-PAX3::Foxo1; Trp53^−/−^	Differentiating myoblasts	Multifocal trunk and limb tumors with alveolar histology	[41]
		Stk3^F/F^; Stk4^PF/PF^; Pax3^PF/PF^; Cdkn2a^F/F^; Myf6^ICN/+^	Differentiating myoblasts	Multifocal tumors with ARMS histology occurring predominantly in the head and neck	[48]
		*aP2-Cre; Cdkn2a^Flox/Flox^; Pax3^P3Fm/P3Fm^; R26-tdTom*	Endothelial	Exclusive to head and neck	[27]
	Zebrafish	CMV-GFP2A-PAX3FOXO1; tp53^M214K/M214K^	Unknown	Tumors with ARMS histology in the caudal fin musculature	[29]
	Drosophila	Mhc-Gal4; UAS-PAX7::Foxo1	Syncytial myofibers	Nucleated cells that spread to larval central nervous system	[76]
SCRMS	Zebrafish	CMV-GFP2A-VGLL2::NCOA2	Unknown	Tumors originating in the tail, head, back, and occasionally ventral side with differing histology by site	[77,78]

Cell-of-origin information for many models adapted from [79]. RMS: rhabdomyosarcoma; FN: fusion-negative; FP: fusion-positive; SCRMS: spindle cell/sclerosing rhabdomyosarcoma.

### 3.2. Hippo Pathway Models

One emerging target for combination with RAS pathway-directed agents is YAP1, a transcriptional co-activator that is negatively regulated by the Hippo pathway [80,81]. YAP1 is activated in RMS patient samples and is an established driver in both FP-RMS and FN-RMS [68,82,83]. TAZ (gene name *WWTR1*), a YAP1 homolog, is also implicated in FP-RMS tumorigenesis [84], and high expression and nuclear localization have been associated with poor survival in FN-RMS [85]. In addition to promoting proliferation and blocking myogenic differentiation, YAP1 activation has been shown to overcome oncogene-induced senescence associated with *Pax3::Foxo1* fusion oncogene and *Ras* oncogene expression in both GEMM and cell line models [48,68,82]. While activating point mutations have not been described in human samples, *YAP1* overexpression and copy number amplification has been observed [82,83]. Current animal models, however, frequently rely on YAP1 S127A mutations or loss of upstream Hippo pathway effectors that negatively regulate YAP1 activity, such as the kinase LATS1 [48,83]. Genetically engineered animal models of TAZ have not yet been described for RMS. Multi-omics technologies should be utilized to ensure that the profiles associated with YAP1 overexpression or amplification in human samples match those of the YAP1 activating mutations used in available cell and animal models. More recent advances in developing YAP-targeting agents necessitate that RMS models faithfully recapitulate human disease before undergoing preclinical testing. Indeed, several novel YAP1-targeting therapeutics are being tested in clinical trials, and their preclinical evaluation of activity in relevant RMS models should be pursued [84].

### 3.3. PAX3::FOXO1 Fusion Oncogene Models

*PAX3::FOXO1*-driven RMS is associated with a poor outcome, and considerable effort has been made to model this subtype. As discussed previously, GEMMs of FP-RMS have been pivotal to the current understanding of tumorigenesis and the possible cells of origin [27,41,42,43,45,62]. However, therapies specifically targeting FP-RMS have yet to show benefit in the clinic, despite some promising preclinical studies [86,87,88]. Improving FP-RMS models to better resemble human disease may improve the successful translation of FP-targeting agents. One way to improve FP-RMS models is by accurately representing co-occurring genetic lesions.

While senescence is bypassed in *PAX3::FOXO1*-expressing human skeletal muscle precursor cells by spontaneous promoter methylation and subsequent down-regulation of p16^INK4A^, oncogene-induced senescence is commonly observed in *PAX3::FOXO1*-expressing FP-RMS animal models [89]. Thus, in addition to Hippo pathway suppression discussed previously, *Tp53* or *Cdkn2A* loss has been used to overcome this phenomenon in preclinical models [9,27,41,42]. However, the somatic mutation burden of *TP53* or *CDKN2A* in FP-RMS patient samples is low and *PAX3/7::FOXO1* fusions commonly occur in isolation [13,58]. Still, these models are incredibly important because co-occurring *TP53* mutation, while very rare (5/126), was universally fatal in a cohort of FP-RMS tumors [58]. FP-RMS models with co-occurring *Tp53* loss or alteration replicate an aggressive but rare molecular subtype, and there is a need for a more broadly applicable model. For example, the most common co-occurring lesion in FP-RMS tumors is amplification of 12q13-q14, which is associated with amplification of genes including *CDK4* and *6* and *SHMT2* [58,90], but these genetic lesions have yet to be modeled. iPSC models like those described previously [27] offer a high-throughput and cell-of-origin agnostic method to study these co-occurring lesions. A more complete modeling of co-occurring genetic lesions could lead to an improved understanding of FP-RMS biology, predict responses to specific therapies, and potentially reveal new targets for this difficult-to-treat subtype.

### 3.4. Reflecting Heterogeneity in Patient Demographics

Diversity in genetic lesions is one source of heterogeneity in RMS. Additional under-explored areas hampering effective modeling of RMS include sex- and age-dependent differences in tumor growth and therapeutic response. RMS incidence is higher in males than females [3], but the influence of sex on tumor development is not well studied. In a GEMM of FN-RMS with Nf1 and Ink4a/Arf loss, investigators observed reduced tumor implantation and growth in female mice compared to male mice [67]. Interestingly, the overall incidence of NF1-associated tumors is higher in females than males for pediatric patients [91]. Additional studies are needed to determine if sex influences RMS development and therapeutic response.

While RMS predominantly occurs in pediatric patients, the range of age at diagnosis is broad. The age of disease onset is a source of patient heterogeneity that is not yet recapitulated in models of RMS, but it is a known prognostic feature. Patients diagnosed in infancy (<1 year) or over the age of ten have worse outcomes [52]. While many factors, such as subtype prominence at these ages and tolerability of aggressive therapy, may contribute to outcome variations, differences in developmental programming of cells in individuals at different ages may also contribute. For example, in the *Ptch^+/−^* mouse model of ERMS, oncogenic Nras expression accelerates tumor penetrance and growth when initiated in two-week-old mice, yet in four-week-old *Ptch^+/−^* mice, oncogenic Nras expression induces tumor differentiation [65,66]. Age at oncogene induction in mouse models may also affect sarcomagenesis. For example, PAX3::FOXO1 could more efficiently generate RMS tumors in postnatal-day-30 mice than in postnatal-day-0 pups [92]. While MyoD-Cre-driven expression of *Kras^G12D^* and *Tp53* loss induces undifferentiated pleomorphic sarcoma-like tumors when induced in adult mice [62], the same model, when induced in utero, produces FN-RMS-like tumors with high homology to human disease [63].

In addition to the tumor-intrinsic effects of age, the age of stromal cells in the tumor microenvironment (TME) may affect RMS progression. In several adult cancers, an aged stromal microenvironment is associated with disease progression and poorer outcomes [93]. In osteosarcoma [94], enrichment of age-related gene expression is associated with reduced immune cell infiltration and poor outcome. However, the contribution of the developing TME to RMS tumor progression is currently unknown, and studies utilizing mice at ages more reflective of human patients are needed. A better understanding of the effect of age or developmental stage on tumorigenesis and progression may reveal therapeutic vulnerabilities, especially for higher-risk patient age groups. With the availability of embryo-based RMS models [30,95,96,97] and PDX models that reflect heterogeneity in patient age, this may be a promising future direction for the RMS field.

### 3.5. Modeling RMS Heterogeneity with Xenografts and Organoid Models 

**Figure 1:**
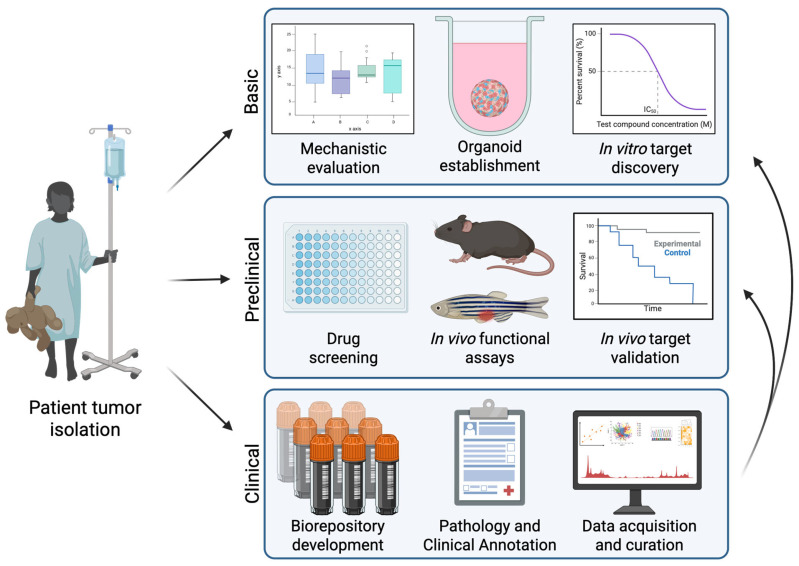
Utilization of patient and patient-derived tumors to recapitulate RMS heterogeneity. Patient samples and patient-derived resources drive discovery in the RMS field, from basic studies of RMS biology, to preclinical evaluation of novel therapeutic strategies or improved prognostic parameters, to the development of biorepositories with clinically annotated samples and datasets. When well curated and appropriately accessible, these biorepositories can continuously support additional basic and preclinical studies. The support and growth of these resources are essential to accurately modeling RMS heterogeneity. Created in BioRender. Hebron, K. (2026) https://BioRender.com/3o7qolw, accessed on 26 February 2026.

Although genetically engineered animal models have advantages such as intact immune systems and tumor–host species concordance, these models cannot reflect the full heterogeneity, both inter- and intratumoral, of human RMS. Several therapeutics showing preclinical efficacy are genotype-specific, so modeling intertumoral heterogeneity is critical [54,55,56,72,98,99]. Additionally, while transcriptomic and histologic analysis indicate that these models closely resemble human RMS, the fact remains that they are murine or zebrafish in origin, which may limit their translatability [51]. Cell line- and patient-derived xenograft (CDX and PDX, respectively) models represent additional tools that complement genetically engineered models (Figure 1, Table 3, and Supplemental Table S1). For example, no genetically engineered models exist that replicate the human RMS condition of co-occurring *RAS* mutations and loss of *NF1* or *BCOR* tumor suppressors, but there are PDX models with this molecular profile [54]. In fact, there is a growing collection of RMS PDXs that have been developed by institutions and investigators, including an extensive collection of models that are freely available to investigators through the Childhood Solid Tumor Network (CSTN) at St. Jude Research Hospital (Tennessee, USA). The CSTN has amassed 47 rhabdomyosarcoma orthotopic-PDX (O-PDX) models representing a range of subtypes, mutations, and patient demographics (as of January 2025) [100]. Many of these samples are available with annotated genetic, transcriptomic (bulk and single-cell), epigenetic, histological, and clinical data. Similarly, the nascent Pediatric Research in Oncology Xenografting Consortium (PROXC) has a federated collection of >500 PDX models developed at six participating centers with expertise in PDX development and preclinical drug testing (including St. Jude) and it includes >50 additional RMS PDX models (https://www.mskcc.org/teaser/proxc-pdx-models-search-portal, accessed on 26 February 2026). These invaluable resources are readily available to the RMS research community to be utilized in the preclinical evaluation of novel therapies. While PDX-based mouse studies can be resource-intensive, strategies such as single-mouse xenograft assays, which predict response with 78% accuracy compared to the response in a group of mice, can be utilized to assess the effects of therapeutics across a molecularly diverse panel of CDX and PDX models in a more cost-effective way than traditional murine studies [50,101].

In addition to CDX and PDX mouse models, zebrafish represent another potential model system for RMS xenografting that can be leveraged to do high-throughput assays for both preclinical and basic studies. Optically clear, immunodeficient zebrafish (*prkdc^−/−^, il2rgs^−/−^*) are capable of engrafting CDXs and PDXs, including RMS, and may aid in identifying molecular subgroups that are more or less likely to respond to specific therapies [102]. Zebrafish embryos with RMS xenografts are also valuable for large-scale drug screening, adding a rapid and cost-efficient model system to the RMS research toolbox [95,97]. Using a *flk1::GFP* transgene strain to label endothelial cells, a recent study demonstrated the value of embryo zebrafish in not only assessing xenograft response to drugs but also in assessing the impact of different xenografts on induction of neovascularization [103]. The vessel label allowed researchers to assess the impact of drugs, with an emphasis on multi-tyrosine kinase inhibitors, on this neovascularization and the interplay of tumor growth and neovascularization [103]. Going forward, comparing different preclinical models amongst each other and in the context of co-clinical trials will further define the place for embryo zebrafish models in RMS research.

### 3.6. Remaining Challenges

RMS models have advanced significantly in their representation of human disease; however, challenges remain, particularly in interlaboratory variability within established models, preserving rare tumor populations from patient tissue, modeling rare RMS subtypes, and profiling the TME. While animal models and patient-derived samples are essential for translational research, some level of reductionism is necessary to understand RMS at the molecular level. In these instances, tissue cell culture has proven invaluable, provided certain factors, such as culture conditions, are considered. Cell culture conditions can create microevolutionary pressures and cause genetic drift in established cell lines [104], as seen in RMS cell lines like RD, where *MYC* amplification varies (personal communication, CML and Dr. Xiang Chen, St. Jude Children’s Research Hospital, 30 May 2025). In this example, altered *MYC* status would be expected to influence the cell line’s biology and therapeutic response; therefore, it is the researcher’s responsibility to verify their resources and continuously monitor for drift to maintain reproducibility and rigor. Moreover, ideal culturing conditions for patient-derived tissue have yet to be defined. For example, some aspects of intratumoral heterogeneity, especially those driving cell state plasticity, may be lost upon 2D culturing of primary patient or PDX tumors [31,32], while 3D culture may enrich subpopulation heterogeneity [105,106,107]. Drug response, for example with RAS pathway inhibitors, may also differ in 2D versus 3D cell culture conditions [108]. Therefore, 3D culture approaches that preserve rare tumor subpopulations and better assess therapeutic response should be incorporated into drug screening and basic mechanistic studies. Additional investigations into culture conditions that preserve rare cell populations are necessary, especially considering that these rare cell types may be progenitor cells that represent more resistant populations and contribute to clinical treatment failure.

Promising developments have been made in modeling less common RMS genotypes. Functional genomics screens in zebrafish were used to investigate rare fusions such as VGLL2::NCOA2, which is associated with a subset of infantile spindle cell RMS [59,60]. However, models of rare and ultra-rare RMS subtypes are less common. The only genetically engineered *PAX7::FOXO1* fusion oncogene model is in *Drosophila*, which is valuable for basic biological studies but limited for translational studies [76]. Notably, there are no genetically engineered animal models of MYOD1^L122R^ RMS, an ultra-rare but highly lethal subtype of RMS; cell lines and PDX models are limited for this entity. To date, most studies have utilized ectopic expression of MYOD1^L122R^ in myoblasts to investigate the biology of MYOD1^L122R^ RMS [109,110]. Considering the extremely poor prognosis of patients with MYOD1^L122R^ RMS, this represents an unmet need in the RMS field.

The influence of the TME on RMS progression and therapeutic response is poorly understood. RMS tumors are generally considered immunologically cold, but profiling of the RMS TME has been limited thus far [34]. With immune checkpoint inhibitors showing little promise in clinical trials including RMS patients [111], a comprehensive understanding of the RMS immune and non-immune TME is needed. Additionally, a rigorous comparison of the human RMS TME and animal models of RMS will improve model selection for preclinical studies. While the collective effort to model RMS (Table 2 and Table 3) has made significant progress, it is clear that it will be important to carefully consider experimental conditions (e.g., 2D versus 3D culture) and model selection to accurately capture the heterogeneity of RMS in the laboratory setting. Continued model and resource development will be critical to help the field rigorously assess novel agents and effectively translate findings.

**Table 3:** Established RMS cell lines.

Subtype	Cell Line	Origin	Known Mutations	Ref.
FN-RMS	CCA	Recurrence, “vesical”	KRAS, p.Gln61Leu	[112]
	CT-TC	Primary	HRAS, p.Gln61Lys	[113]
	FL-OH1	Pediatric M, primary	XY, many clonal and non-clonal aberrations, negative for t(1;13), t(2;13)	[114]
	HX170c	5yo M, recurrence, paratesticular	NRAS, p.Gln61Lys; PIK3CA, p.Glu545Lys	[115]
	Hs 729.T	74yo M	TP53, p.His179Arg	
	JR-1	7yo F, metastasis, lung	NRAS, p.Gln61Lys; TP53, p.Arg249Ser	[116]
	KF-RMS-1	Metastasis, bone marrow	unknown	[117]
	RD	7yo F, primary, pelvic mass	NRAS, p.Gln61His; TP53, p.Arg248Trp	[118]
	RH2	Unknown	IGF1R, p.Pro27Ser	
	RH6	16yo M, metastasis, lymph node	unknown	
	RH12	Buttock	unknown	[119]
	RH14	Recurrence, inguinal	unknown	[120]
	RH18 FN	Unknown	Amplification 12q13–15 including MDM2; IGF2R, p. Gly1860Asp	[89,119,120]
	RH36 (Birch)	15yo M, relapse, paratesticular	HRAS, p.Gln61Lys	[121]
	RMS559	M, age unspecified	FGFR4, p.Val550Leu; PTPN11, p.Glu68Lys	[122]
	RMS-YM	2yo M, abdominal	BCOR, p.Arg1163Ter	[123]
	RUCH2	15-month-old F, vaginal, botryoid variant	BRAF, p.Val600Glu	[124]
	RUCH3	M, relapse	TP53, p.His193Arg	[124]
	SMS-CTR	1yo M, pelvic	HRAS, p.Gln61Lys; TP53, p.Glu221Gln, p.Glu221Lys, p.Glu221Ter	[125]
	TTC442	10yo M	TP53, p.Arg248Gln	[126]
	TTC516	12yo M	IGF1R, p.Lys560Arg; KMT2A, p.Glu2989Lys	[126]
	DI-OH1	Pediatric M, primary	unknown	[114]
	VK	20yo F, localized primary	unknown	[127]
	RMS-GR	75yo M, relapse, urogenital	unknown	[128]
	TS-RM-1	Pediatric F	unknown	[129]
	YN	15yo M, paratesticular	unknown	[130]
	TE617T	20-month-old F	unknown	
	JH-ERMS-1	10yo, metastasis	HRAS, p.Gly12C; NF1, p.W336, p.N441fs; BCOR, p.L1375fs; TP53, p.L111R; TSC2, p.L1754M; MET, p.S156L	[54]
	JH-ERMS-2	5yo, recurrence, orbital	HRAS, p.Gly12Ser; PIK3CA, p.Ala546Glu	[54]
	KMR19	Murine GEMM-derived	Kras^G12D^; Tp53^R172H/+^	[63]
	KMR46	Murine GEMM-derived	Kras^G12D^; Tp53^F/+^	[63]
	KMR72	Murine GEMM-derived	Kras^G12D^; Tp53^R172H/+^	[63]
	KMR78	Murine GEMM-derived	Kras^G12D^; Tp53^R172H/+^	[63]
	Kras-H myoblasts	Murine p53-deficient myoblasts	Trp53^−/−^; KRASp.Gly12Asp	[39]
	M25.FGFR4 (V550E)	Murine p53-deficient myoblasts	Trp53^−/−^; FGFR4, p.Val550Glu	[131]
	MY-THR	Primary human myoblasts (HSMM)	T/t-Ag; hTERT; HRAS, p.Gly12Val	[40]
FP-RMS	CW9019	Unknown	PAX7::FOXO1	[120]
	D-RHA1	Unknown	Unknown	[126]
	KFR	13yo F, metastasis, bone marrow	Pseudodiploid, PAX3::FOXO1, (q35;q14)	[132]
	RH5	Unknown	PAX3::FOXO1; TP53, p. Arg248Gln	[133]
	RH10	15yo F, relapse, perineal	Hyperdiploid, t(2;13)	[134]
	RH18 FP	2yo F, perineal	PAX3::FOXO1	[119,120,135]
	RH28 (RH3)	17yo M, metastasis, axillary	Near tetraploid, PAX3::FOXO1	[134]
	RH30	16yo M, metastasis, bone marrow	PAX3::FOXO1; TP53, p.Arg273Cys; amplification 12q13–15 including *CDK4*	
	RH41 (RH4)	7yo F, metastasis, lung	PAX3::FOXO1	[112]
	RH65	18yo F, relapse	Unknown	[120]
	RMZ-RC2 (RC2)	2yo M, metastasis, bone marrow	PAX7::FOXO1, *NMYC* amplification	[136]
	TC212	16yo M, metastasis, bone marrow	t(2;13)(q35;q14)	[125]
	CB-NJR	Unknown	Unknown	[137]
	HA-OH1	Pediatric F, relapse	XXX, der(1), t(1;13), der(1), t(1;13), i(q), del(3), 4q+, del(6), −13, [cp4]	[114]
	HUMEMS	13yo F, primary, breast (malignant pleural effusion)	PAX3::FOXO1	[138]
	NRS-1	7yo F, primary, forearm	PAX3::FOXO1	[139]
	RH7	Primary	PAX3::FOXO1	[113]
	UISO-RS-3	28yo F, primary, buttock (malignant pleural effusion)	X, Philadelphia 22 chromosome (70% of metaphase spreads)	[140]
	Not named	14yo F, primary, chest wall (malignant pleural effusion)	t(2,13)(q37;q14), double minute chromosomes	[141]
	JR	Unknown	PAX3::FOXO1; TP53, p.Thr377Pro	
	SCMC-RM2	11yo F, metastasis, bone marrow	PAX3::FOXO1; DICER1, p.Ser678Phe	
SCRMS	C2C12-VGLL2::NCOA2	Immortalized murine myoblasts	VGLL2::NCOA2	[77]
	TCCC-ST78	Unknown	MYOD1, p.Lys122Arg	

Where available, origin column lists patient age and sex, disease instance, and tumor location. Mutations are listed as described in the publication initially characterizing the cell line. RMS: rhabdomyosarcoma; FN: fusion-negative; FP: fusion-positive; SCRMS: spindle cell/sclerosing rhabdomyosarcoma; yo: years old; F: female; M: male. Please see Supplemental Table S1 for Cellosaurus and DepMap identifiers for these cell lines.

## 4. How Can Sequencing Technologies Be Leveraged to Gain a Better Understanding of RMS Biology and Predict Clinical Response to Therapies?

Research on the genetic underpinnings of RMS has identified critical genetic drivers associated with histological subtypes. Advances in next-generation sequencing (NGS) have made it more feasible to perform rapid, unbiased studies of genomic changes in tumor samples. This has provided novel insights for future mechanistic studies and led to the development of novel diagnostic and prognostic tools. In addition to genomic discoveries, NGS efforts illustrate that epigenetic dysregulation occurs frequently in RMS. Here, we will discuss aspects of the knowledge that may be gained from sequencing technologies.

### 4.1. Unbiased and Comprehensive Detection of RMS Genetic Abnormalities

An early collaborative effort between the National Cancer Institute (NCI), Children’s Oncology Group (COG), and the Broad Institute used a multi-omics approach to identify recurrent alterations in a cohort of patients with RMS. WGS (whole-genome sequencing), WES (whole-exome sequencing), and WTS (whole-transcriptome sequencing) were performed on 147 RMS patient samples. A novel fusion event, *PAX3::INO80D*, and somatic mutations not previously reported or studied in RMS, such as *FBXW7*, *BCOR*, *CTNNB1*, *FOXO1*, and *ARID1A*, were found in this cohort [13]. Moreover, copy number variations (CNVs) were identified in genes commonly involved in dysregulated proliferation, apoptosis, and myogenic differentiation signaling pathways, such as *CDK4*, *FRS2*, *MDM2*, *MYCN*, and *CDKN2A* [13]. In a more recent consortium effort aimed at using genomic datasets to predict patient outcomes [58], the COG and the UK Institute of Cancer Research reported that *TP53* and *MYOD1*^L122R^ mutations are associated with poor survival rates, even in patients with otherwise low-risk clinical features, based on data from 641 patients diagnosed between 1995 and 2017 [58] (data available through the NCI ClinOmics dataset). These findings have been incorporated into risk stratification in ongoing RMS clinical trials (NCT05304585 and NCT06023641). Interestingly, despite previous indication that RAS pathway mutations were a poor prognostic marker in FN-RMS [60], this study found no impact on outcome [58]. Meanwhile, another report showed that patients with a germline mutation in *HRAS* had significantly worse outcomes than those with no germline mutations [142]. These results highlight that there are open questions regarding the role of RAS pathway mutations in the prognostication of FN-RMS.

In relation to germline genetics, another report identified pathogenic germline variants in a cohort (273 for discovery; 121 for validation) of patients with RMS [143]. This assessment explored the frequency of germline pathogenic/likely pathogenic variants in cancer predisposition genes (CPGs), including *TP53*, *NF1*, *DICER1*, *BRCA2*, *CBL*, *CHEK2*, and *SMARCA4*. In a companion study leveraging data on 615 patients enrolled in COG protocols, investigators found that 7.3% of those with RMS harbored a pathogenic/likely pathogenic variant in established CPGs [144]. However, the prevalence of these variants was significantly different based on age of diagnosis, histology, and fusion status. Finally, there is emerging evidence that variants in CPGs and common germline variants are associated with survival outcomes [142,145].

These cooperative efforts highlight the vital application of sequencing technologies to identify recurrent molecular features with clinical significance. With this new knowledge, a next step is to characterize the effect of these lesions on cellular functions. While many of the identified alterations have been described in other tumor types, their effect on RMS tumor pathology, such as oncogenesis, aggressiveness, or response to therapy, and cancer cell behavior, such as proliferation, differentiation, and survival, have not been fully elucidated. Most importantly, we must understand how the information collected from individual patients can be used to predict responses to specific drugs and radiotherapy and to identify additional targets for therapeutic pharmacological exploitation that are downstream of the observed genetic mutations.

### 4.2. Non-Invasive Sequencing Approaches in Risk Stratification and Therapeutic Response

Advances in technologies such as circulating tumor DNA (ctDNA) extraction and sequencing with high sensitivity demonstrate how sequencing technology may be used to identify therapeutic vulnerabilities and predict patient outcomes [146,147]. For example, researchers have begun to identify evolving genetic mutations in RMS via liquid biopsies where ctDNA from patient blood draws has been extracted and sequenced over serial timepoints [148]. The prognostic value of ctDNA levels at certain points in therapy has also been demonstrated. Specifically, newly diagnosed patients with intermediate-risk FN-RMS who had detectable levels of ctDNA at diagnosis had significantly worse outcomes than those without detectable ctDNA [149]. Similarly, in a clinical trial assessing ganitumab (a monoclonal antibody targeting the type 1 insulin-like growth factor receptor) and dasatinib (multi-target tyrosine kinase inhibitor) in patients with relapsed RMS, an early reduction in the presence of cell-free DNA (cfDNA) correlated with better responses to these drugs [98]. These studies highlight the potential uses of ctDNA and cfDNA as an effective and non-invasive method to improve risk stratification, evaluate responses to pharmacologic agents, and follow molecular changes in RMS in patients over time.

### 4.3. Identifying Epigenetic Aberrations in RMS

Epigenetic sequencing has increased our knowledge of RMS biology and has identified predictors of response to therapy [150]. In recent decades, with the advent of DNA methylation sequencing techniques, DNA methylation profiles of patient samples have been increasingly used to classify patient tumors into molecular subgroups [151]. Combining bisulfite sequencing with single-cell technology, researchers can profile “epimutations” of patient tumors at the single-cell level, supplementing information gleaned from single-cell transcriptomics.

Chromatin immunoprecipitation sequencing (ChIPseq) has identified chromatin structure alterations that contribute to RMS development and have potential therapeutic implications [152,153,154]. EZH2 is an enzyme within the polycomb repressor complex 2, which suppresses gene expression by trimethylating histone H3 on lysine 27 and compacting proximal chromatin. This mechanism maintains RMS cells in an indolent and less differentiated cell state, which is less responsive to conventional therapies [152,153,154]. In contrast, KDM3A and KDM3B, two histone demythelases, were shown to promote the transcriptional activation function of PAX3::FOXO1 fusion oncoproteins by altering the histone modification and chromatin accessibility in RMS cells [155,156,157,158]. These findings provide implications of cancer cell vulnerability by targeting these KDM proteins rather than the challenging target of fusion protein itself. In addition, histone deacetylases (HDACs) regulate chromatin condensation by removing acetyl groups from histones and activating the transcription of oncogenes or genes that promote RMS proliferation. CRISPR- and drug-based screening in vitro have identified HDAC3 as an important suppressor of myogenic differentiation in RMS [159] and promoter of radioresistance [160], illuminating the potential contribution of epigenetic sequencing efforts in therapy development. Additionally, HDAC6 has been shown to regulate migration, invasion, and self-renewal in FN-RMS through the Rho family GTPase, RAC1, identifying an additional targetable epigenetic modulator [161]. Chemical genomics approaches have revealed that, in *PAX3::FOXO1*-driven RMS, HDAC1/2 is a critical dependency, actionable through HDAC inhibitors such as Entinostat that selectively downregulate essential transcription factors (MYOD1, MYCN, SOX8) [162,163].

**Figure 2:**
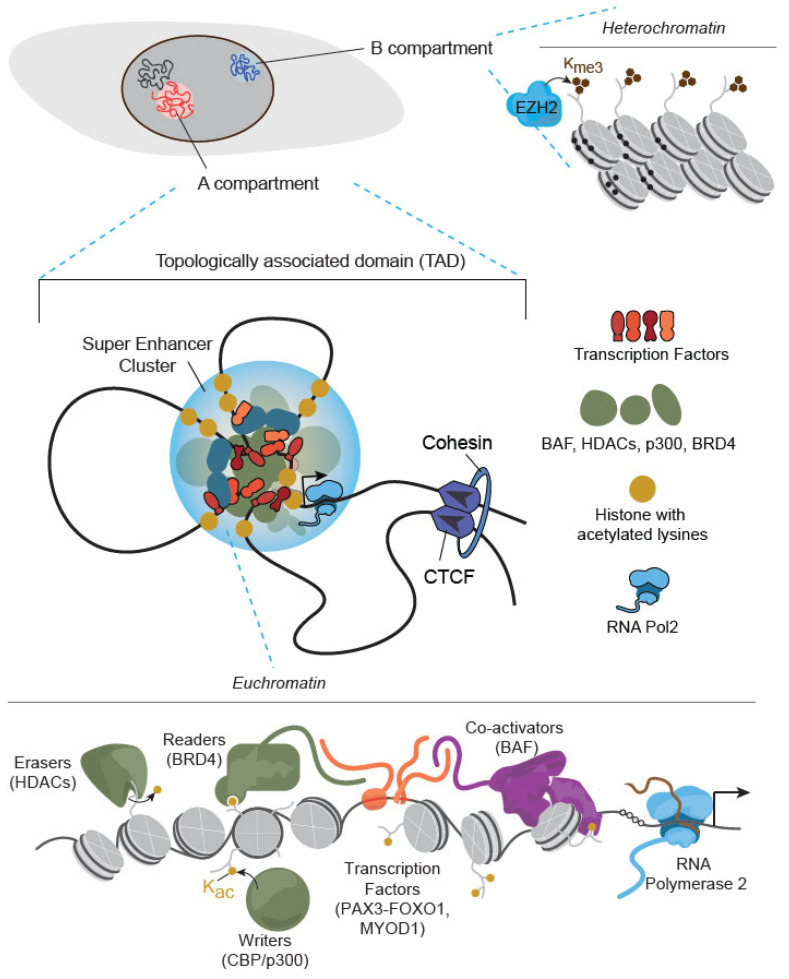
Chromatin structure, transcription factors and epigenetic co-factors dysregulated in RMS. Top: Genomic information is spatially segregated into active “A” compartment (euchromatin) and suppressive “B” compartments (heterochromatin). Heterochromatin regions are silenced and marked by histone lysine (K) tri-methylation (me3), mediated by PRC2 complex with the methyltransferase enzyme EZH2. In RMS, EZH2 shuts down myogenic differentiation genes, locking cells in an undifferentiated state. Middle: In euchromatin regions at key RMS-driven genes, transcriptional machinery is organized into super-enhancer clusters within topologically associated domains (TADs). These genes are driven by myogenic transcriptional factors, such as MYOD1 and MYOG, and chromatin modifiers like BAF, HDACs, p300, and BRD4, which recruit and activate RNA polymerase II. Bottom: Unbiased drug screening has identified the vulnerability of RMS cells to HDACs or CBP/p300 inhibitors due to their critical roles in aberrant transcriptional activation. Additionally, PAX3-FOXO1, mutant MYOD1, and other transcription factors such as NF-Y have been shown to hijack the existing transcriptional machinery, re-wiring muscle-lineage epigenetic programs to establish RMS.

As knowledge of epigenetic modifiers accumulates, there has been an increase in the understanding of how myogenic regulators, such as *MYOD1* and *PAX3/PAX7::FOXO1* oncogenes, depend on other protein complexes to perform their transcription factor functions. For example, MYOD1 recruits BRG1-associated BAF complex to drive muscle commitment and differentiation [164]. A central question in the field is how PAX3::FOXO1 initially engages chromatin. Recent studies reveal that PAX3::FOXO1 can engage multiple chromatin states to facilitate DNA accessibility transitions [86,165,166]. New approaches have been developed to study and map the 3D chromatin state in rhabdomyosarcoma [167,168] with recent creative adaptations to obtain highly quantitative measurements of PAX3::FOXO1 genome binding [168]. New studies emerging will begin to integrate dosage-dependent mechanisms for PAX3::FOXO1’s molecular recognition and restructuring of the epigenome and investigate how this may cause transcriptional changes, giving rise to the major cell subpopulations found in human RMS tumors [35,169]. PAX3::FOXO1 acts as a super-enhancer regulator that depends on BRD4 to open chromatin and regulate critical signals for cells to maintain the undifferentiated cell state [86,163], and BRD4 inhibition can slow the growth of ARMS in PDX models [170]. Genetic and molecular dissection of PAX3::FOXO1 has revealed that CBP/p300 are also directly recruited by the fusion oncogene, setting up expansive RNA Polymerase IIclusters at key myogenic and pro-proliferative genes [171]. Transcription factors SIX1 and SNAI2 compete for chromatin binding with MYOD1 at MYOD1binding E-Boxes to inhibit differentiation in RMS [172,173,174]. SKP2, an E3 ubiquitin ligase, is another RMS oncogene that blocks myogenic differentiation by directly targeting p27 and p57 for degradation [174]. Functional CRISPR screening in FP-RMS has revealed that Nuclear Factor Y (NF-Y) blocks myogenic differentiation by transcriptionally activating *PAX3::FOXO1* expression [175]. Figure 2 summarizes the mechanisms involved in the epigenetic modification of RMS, with a special focus on chromatin structure and the critical transcription factors whose dysregulation plays a pivotal role in mediating the aggressive phenotype of RMS cells.

Together, these studies highlight a complex network of interacting proteins that reprogram the cellular epigenetic landscape and transcriptome, conferring an undifferentiated cell state with unlimited proliferation potential on RMS. Future evaluations of how these epigenetic changes can be employed for prognostic purposes or therapeutic targeting are underway (NCT04705818). With an increased understanding of RMS genomics and epigenomics, there will be increased opportunities to develop novel therapeutic strategies to target these potential secondary drivers in RMS.

## 5. What Are the Challenges Impeding the Clinical Translation of Basic Discoveries in RMS, and How Can They Be Addressed?

Despite an increase in the identification of potentially actionable targets in RMS, efforts to translate targeted therapeutics have been stymied by several challenges. The translation of cancer therapeutics from the bench to the clinic is arduous, and most investigational cancer drugs fail to demonstrate sufficient success to attain drug approval [176]. Factors contributing to this failure are frequently related to the differences between preclinical models and patients, including differences in species tolerance to drugs, tumor models that do not recapitulate human cancer at the molecular/genetic level and do not encompass clinical heterogeneity, difficulties in defining activity in preclinical models, unforeseen toxicities not noted in preclinical models, and the lack of preclinical models that include a functional immune system. Further, pediatric clinical trials lag behind adult trials, and negative results in adult trials often lead to drug discontinuation before adequate testing in pediatric indications.

Additional roadblocks for RMS specifically include a small patient population that limits the potential financial return of RMS-specific drugs. This leads to “financial orphan” drugs that may show activity in preclinical or early-phase clinical trials but lack a path forward due to the absence of industry support [72,98,177,178]. For example, despite promising results in a Phase I trial of the combination of ganitumab and dasatinib in patients with relapsed or refractory RMS, a patient population with very limited therapeutic options, Phase II enrollment was halted due to loss of access to ganitumab [98]. Thus, there is a critical need for sustained partnerships with industry sponsors to ensure the continued translation of promising novel therapeutics for RMS. Encouragingly, legislation has been passed to facilitate and incentivize drug development for pediatric populations. In the United States (U.S.), the Research to Accelerate Cures and Equity (RACE) for Children Act (H.R. 1231) authorizes the U.S. Food and Drug Administration (FDA) to require that companies include pediatric populations in clinical trials with a relevant molecular target. Early evidence suggests that this legislation has effectively increased the number of clinical trials that include pediatric populations. Still, it is too early to determine whether this will translate to new therapies for pediatric cancer patients, specifically for those who have cancers not widely represented in the adult population [179]. International efforts to coordinate an ongoing concerted effort between patient advocacy groups, researchers, and legislators will be required to ensure pediatric-specific therapies are developed. One such international effort, ACCELERATE, coordinates the equal involvement of these stakeholder groups (researchers, patient advocates, industry partners, and regulators) to facilitate patient-centered childhood cancer drug development (https://www.accelerate-platform.org, accessed on 26 February 2026). In addition, the U.S. FDA, in collaboration with the National Center for Advancing Translational Science (National Institutes of Health), have developed CURE ID (https://cure.ncats.io/home, accessed on 26 February 2026), an internet-based repository that lets patients, caregivers, and the clinical community report novel uses of existing drugs for difficult-to-treat diseases such as rare sarcomas through a website, a smartphone or other mobile device. The platform enables the crowdsourcing of medical information to facilitate the development of new treatments for these orphaned diseases.

The small patient population also limits the number of tissue samples available for research. To maximize the value of this precious resource, data acquisition and distribution should be standardized. Several national and international efforts are being undertaken to address this problem (Table 4). For example, the National Cancer Institute’s Childhood Cancer Data Initiative (CCDI) Molecular Characterization Initiative [180] and the Children’s Oncology Group’s Project:EveryChild (https://childrensoncologygroup.org/cog-registry-project-everychild, accessed on 26 February 2026) seek to establish comprehensive databases of molecular and clinical data from pediatric cancer patients with long-term follow-up. Resources such as the R2 Genomics Analysis and Visualization Platform (http://r2.amc.nl, accessed on 26 February 2026) and the ClinOmics database (https://clinomics.ccr.cancer.gov/clinomics/public/, accessed on 26 February 2026) compile published -omics datasets with user-friendly visualization tools. International efforts such as INSTRuCT (International Soft Tissue Sarcoma Consortium) are underway to standardize clinical trial data collection and imaging protocols [181,182,183] and to make valuable imaging and pathology data accessible to researchers. Research autopsy programs, which perform comprehensive sample collection within a short postmortem timeframe, are another potential mechanism for overcoming the hurdle of limited patient samples [184]. They have contributed to significant advances in understanding metastatic progression [185], disease heterogeneity [186], therapeutic response, and resistance [187]. Careful consideration of the unique challenges of implementing rapid autopsy programs in pediatric populations is essential and will require significant input from patient advocacy groups.

## 6. Conclusions

Continued coordinated efforts between basic scientists, clinicians, industry, and patient advocacy groups are necessary to strengthen the pipeline of therapeutics for RMS. As highlighted here, several focus areas require additional efforts to improve RMS patient outcomes. A more complete understanding of RMS biology, including cell(s) of origin, heterogeneity, the use of a variety of preclinical models with high fidelity to the clinical entities, and standardized data acquisition and accessibility are all potentially promising means to overcome the current roadblocks in translating RMS research discoveries to the clinic and affecting outcomes for patients with RMS. Finally, coordinated, international efforts across rhabdomyosarcoma research and the clinical enterprise, as evidenced in the writing of this manuscript, will continue to be vital to the advancement of RMS treatment.

## Figures and Tables

**Table 1:** Characteristics of the three common pediatric rhabdomyosarcoma histological subtypes.

Pediatric RMS Subtypes	ARMS	ERMS	SCRMS	
**Prevalence**	~20–30%	~60–70%	~10%	
**Histology**	Round cells clustered into nests separated by fibrous septa	Variable: small round cells and large polygonal cells with cross-striations	Spindle cells in a densely hyalinized collagen	
**Age of Incidence**	Late childhood and adolescence	Bimodal, those aged 0–4 and 14–18 years	Infantile	Young adults
**Molecular alteration**	Commonly PAX3::FOXO1, ~60%, or PAX7::FOXO1, ~20%	Variable, RAS-MEK-ERK dysregulation, PI3K activation /PTEN loss, NF1 alterations	Commonly NCOA2::VGLL2	MYOD1^L122R^
**Outcome**	Poor	Intermediate	Favorable	Extremely poor

RMS: rhabdomyosarcoma; ARMS: alveolar RMS; ERMS: embryonal RMS; SCRMS: spindle cell/sclerosing RMS.

**Table 4:** Data and resource sharing initiatives available to rhabdomyosarcoma researchers.

Websites	Description	Links
Allthatglitters	Gene expression in RMS patient samples in a subtype-specific manner	https://allthatglitters.shinyapps.io/exp_compare/ (accessed on 26 February 2026)
Childhood Cancer Data Initiative	The National Cancer Institute CCDI has the goal of creating a Data Ecosystem that centralizes new and existing clinical, sequencing, and demographic data	https://www.cancer.gov/research/areas/childhood/childhood-cancer-data-initiative/data-ecosystem (accessed on 26 February 2026)
DepMap	Cancer vulnerability studies based on RNAi or CRISPR loss-of-function screens of cancer cell lines (including >10 RMS cell lines)	https://depmap.org/portal/ (accessed on 26 February 2026)
INSTRuCT	Pediatric Data Commons; international cohort of centralized clinical data available to researchers upon project proposal and request	https://portal.pedscommons.org/login (accessed on 26 February 2026)
NIH ClinOmics	Next-generation sequencing datasets covering RNA-seq, WES, and WGS on primary patients or patient-derived cell lines of pediatric cancers	https://clinomics.ccr.cancer.gov/clinomics/public/ (accessed on 26 February 2026)
Project:EveryChild	Children’s Oncology Group registry of clinical, demographic, and epidemiological data associated with bio-banked tumor tissue	https://childrensoncologygroup.org/cog-registry-project-everychild (accessed on 26 February 2026)
R2 Genomics Analysis and Visualization Platform	Compilation of published gene expression datasets by tumor type with accessible analysis and visualization tools	http://r2.amc.nl (accessed on 26 February 2026)
Sarcoma CellMinerCDB	NIH/NCI Center for Cancer Research—publicly accessible sarcoma cell line database	https://discover.nci.nih.gov/rsconnect/SarcomaCellMinerCDB/ (accessed on 26 February 2026)
St. Jude CSTN	Childhood Solid Tumor Network, with publicly available RMS O-PDXs and paired sequencing and histopathology data upon request	https://www.stjude.org/research/why-st-jude/data-tools/childhood-solid-tumor-network.html (accessed on 26 February 2026)

## Data Availability

No new data were created or analyzed in this study. Data sharing is not applicable to this article.

## Supplementary Materials

The following supporting information can be downloaded at: https://www.mdpi.com/article/10.3390/cancers18060888/s1, Table S1: Cellosaurus and DepMap identifiers for cell lines listed in Table 3.

## Abbreviations

The following abbreviations are used in this manuscript:

RMS	Rhabdomyosarcoma
ARMS	Alveolar rhabdomyosarcoma
ERMS	Embryonal rhabdomyosarcoma
SCRMS	Spindle cell/sclerosing rhabdomyosarcoma
PRMS	Pleomorphic rhabdomyosarcoma
FP-RMS	Fusion-positive rhabdomyosarcoma
FN-RMS	Fusion-negative rhabdomyosarcoma
GEMM	Genetically engineered mouse model
MRF	Myogenic regulatory factor
PDX	Patient-derived xenograft
CDX	Cell line-derived xenograft
