# Supplementary material for: Biological Advances and Current Challenges for Pediatric Rhabdomyosarcoma
Source: Cancers (Basel). 2026 Mar 10;18(6):888. doi: 10.3390/cancers18060888 (PMC13023981; doi:10.3390/cancers18060888)
Supplement: Supplementary file 1 [file cancers-18-00888-s001.zip › cancers-4153927-supplementary.pdf]

Subtype	Cell line	Cellosaurus ID	DepMap ID
FN-RMS	CCA	CVCL_N717	
	CT-TC	CVCL_N821	
	FL-OH1	CVCL_S870	
	HX170c	CVCL_N850	
	Hs 729.T	CVCL_0871	
	JR-1	CVCL_J063	
	KF-RMS-1	CVCL_S636	
	RD	CVCL_1649	ACH-000169
	RH2	CVCL_A460	
	RH6	CVCL_S639	
	RH12	CVCL_S638	
	RH14	N/A	
	RH18 fusion-negative	N/A	
	RH36 (Birch)	CVCL_M599	
	RMS559	CVCL_S640	
	RMS-YM	CVCL_A792	ACH-002048
	RUCH2	CVCL_C540	
	RUCH3	CVCL_C541	
	SMS-CTR	CVCL_A770	ACH-001196
	TTC442	CVCL_B255	ACH-001750
	TTC516	CVCL_8004	
	DI-OH1	CVCL_S873	
	VK	CVCL_Y578	
	RMS-GR	CVCL_Y577	
	TS-RM-1	CVCL_W501	
	YN	CVCL_Y579	
	TE617T	CVCL_1755	ACH-000051
	JH-ERMS-1	N/A	
	JH-ERMS-2	N/A	
	KMR19	N/A	

	KMR46	N/A	
	KMR72	N/A	
	KMR78	N/A	
	Kras-H myoblasts	N/A	
	M25.FGFR4 (V550E)	N/A	
	MY-THR	N/A	
FP-RMS	CW9019	N/A	
	D-RHA1	CVCL_S635	
	KFR	CVCL_S637	
	RH5	CVCL_5917	
	RH10	CVCL_8750	
	RH18 fusion-positive	CVCL_1659	
	RH28 (RH3)	CVCL_8752 (CVCL_L415)	
	RH30 (RMS13, SJRH30)	CVCL_0041	ACH-000833
	RH41 (RH4)	CVCL_2176 (CVCL_5916)	
	RH65	N/A	
	RMZ-RC2 (RC2)	CVCL_L510	ACH-001743
	TC212	CVCL_S867	
	CB-NJR	CVCL_Y580	
	HA-OH1	CVCL_S869	
	HUMEMS	CVCL_S872	
	NRS-1	CVCL_4871	
	RH7	CVCL_Y583	
	UISO-RS-3	CVCL_Y581	
	Not named	N/A	
	JR	CVCL_RT33	ACH-001096
	SCMC-RM2	CVCL_A667	
PRMS	HS-RMS-1	CVCL_A791	
	HS-RMS-2	CVCL_Y582	
	NCCPRMS1C1	CVCL_B3TM	ACH-002787

	HUS-2	CVCL_S874	
SCRMS	C2C12-VGLL2::NCOA2	N/A	
	TCCC-ST78	N/A	

Supplemental Table 1. Cellosaurus and DepMap identifiers for cell lines listed in table 3. Common aliases are indicated in parentheses, with corresponding Cellosaurus identifiers also in parentheses, where available.
